# Shen-fu injection alleviates acute renal injury by reducing cytokine levels and modulating apoptosis in a porcine hemorrhagic shock model

**DOI:** 10.1590/ACB360405

**Published:** 2021-05-28

**Authors:** Wei Yuan, JunYuan Wu, Qiang Zhang, Yong Liang, MingQqing Zhang, HongJie Qin, Chun-Sheng Li

**Affiliations:** 1 PhD. Capital Medical University – Beijing ChaoYang Hospital – Department of Emergency – Beijing, China.; 2 PhD. Peking University Third Hospital – Department of Critical Care Medicine – Beijing, China.; 3 Master. Beijing Jishuitan Hospital – Department of Emergency Medicine – Beijing, China.; 4 Master. Capital Medical University – Beijing Luhe Hospital – Department of Emergency Medicine – Beijing, China.

**Keywords:** Shock Hemorrhagic, Acute Kidney Injury, Cytokines, Apoptosis

## Abstract

**Purpose:**

Shen-fu injection (SFI) was used to intervene in the resuscitation of porcine hemorrhagic shock (HS) model to study its protective effects on acute kidney injury.

**Methods:**

After 60 min of HS, 28 animals were randomly assigned into four groups. The groups were as follows: hemorrhagic shock group (HS); HS resuscitation with shed-blood group (HSR); HS resuscitation with shed-blood and SFI (1 mL·kg^–1^) group (HSR-SFI); and the sham operation group (Sham). The bloods were analyzed for serum creatinine (sCr), cystatin C (CysC) and neutrophil gelatinase-associated lipocalin (NGAL). BAX, Bcl-2, and caspase-3 protein expressions by Western blot analysis and immunohistochemical staining. The renal tissues were removed and pathologic changes were observed.

**Results:**

Mean aortic pressure (MAP) in HSR-SFI groups were higher than that in HSR groups after shock. At the 6th hour after shock, the urine volume per hour in the HSR-SFI groups was more than that in the HSR groups. The sCr, NGAL, CysC and cytokine levels of HSR-SFI groups were lower. The Bcl-2 expression was increased in the HSR-SFI groups. The BAX and caspase-3 expressions were reduced. The histopathologic score in the HSR-SFI was lower.

**Conclusions:**

SFI may reduce the risk of acute kidney injury (AKI) following hemorrhagic shock by attenuating systemic inflammatory responses, and regulating the expression of apoptosis-related proteins.

## Introduction

Trauma is the third leading cause of death worldwide, and the leading cause of mortality in people older than 44 years^1^. Secondary organ dysfunction is one of the main causes of trauma deaths. Acute kidney injury (AKI) is the most common complication after trauma with a reported incidence of 50%^2^. Acute kidney injury may be caused by several reasons. Except for decreased renal perfusion that results from hemorrhage, rhabdomyolysis, and systematic inflammation, renal oxidative stress during resuscitation is the critical indicator associated with later AKI^3^^–^ 5. Reducing the incidence of AKI may reduce mortality of trauma patients.

Shen-fu injection (SFI) is traditionally Chinese, its main active ingredients are ginsenosides and aconitine alkaloids and its solvent is 100 mL 5% glucose injection^6^. The ginsenoside scavenges free radicals, inhibiting inflammatory mediators^7^^,^ 8, suppressing cellular apoptosis^9^. This traditional Chinese medicine has been routinely administered to treat septic shock, acute myocardial dysfunction, and chronic congestive heart failure and to boost postoperative recovery^10^^,^ 11. On this basis, the hypothesis is that SFI could protect renal function from circulatory insufficiency induced hemorrhage. Reports on the effect of ginseng on hemorrhagic rat led to the design of this study^12^, and this is the first comparative study of SFI in hemorrhagic shock (HS) porcine model. In this study, SFI was used to intervene in the resuscitation of porcine HS model to study its protective effects on AKI and to explore the mechanism of action.

## Methods

The study experimental protocol was approved by the Committee on the Ethics of Animal Experiments of Capital Medical University (Permit No. 2010-D-013). The animal experiments were in compliance with the Guiding Principles for the Care and Use of Animals expressed in the Declaration of Helsinki^13^.

### Study subject characteristics

Twenty-eight male Beijing Landrace pigs were used for this experiment. The pigs were 12 ± 2 months of age, weighed 28 ± 2 kg, and were provided by a registered laboratory animal center in Beijing, China. The animals were fasted overnight with free access to water before surgery.

### Anesthesia and perioperative management

Initial sedation was induced by intramuscular injection of ketamine (0.5 mg·kg^–1^), followed by intravenous injection of propofol (1 mg·kg^–1^). Then, propofol (9 mg·kg^–1^·h^–1^) and fentanyl (1 μg·kg^–1^·h^–1^) were administered intravenously to maintain the anesthesia and analgesia. A cuffed6.5-mm endotracheal tube was advanced into the trachea.

The pigs were mechanically ventilated with a volume-controlled ventilator (Servo 900c; Siemens, Berlin, Germany) with a tidal volume of 8 mL·kg^–1^, a constant fraction of inspired oxygen of 0.21, and an inspiration/expiration ratio of 1:2 with a positive end-expiratory pressure of 5 cm H_2_O. End-tidal PaCO2 was monitored with an in-line infrared capnograph system (CO_2_SMO Plus monitor; Respironics, Inc, Murrysville, PA, USA). Respiratory frequency was adjusted to maintain end-tidal PaCO2 between 35 and 40 mmHg.

The right femoral artery and right external jugular vein were exposed. A 6F catheter (Edwards Lifesciences, Irvine, CA) was advanced from the right femoral artery into the thoracic aorta to collect arterial blood samples and to measure the mean aortic pressure (MAP) using a pressure transducer (Biosensors International Group, Singapore).

The arterial and central venous catheters were connected to an integrated bedside monitor (PiCCO; Pulsion Medical Systems, Munich, Germany) for continuous hemodynamic monitoring including measure right atrial pressure and cardiac output (CO). All catheters were calibrated before use, and tip positions were confirmed by the presence of pressure traces. The electrocardiograph and all hemodynamic parameters were monitored using a multifunction monitor (M1165; Hewlett Packard Enterprise, Palo Alto, CA).

### Experimental protocol

After surgery, the pigs achieved a stable resting level, and baseline data were recorded. The animal model of HS was adapted according to the previous study^14^. Pigs were rapidly bled via arterial sheath in the inguinal to a mean arterial pressure of 40 mmHg within 10 min and were maintained at 40 ± 3 mmHg for 60 min.

Blood was stored in a blood preservation bag (S-400, Sichuang Nightingale Biological Co. Ltd). Additional blood was withdrawn at a mean arterial pressure of 44 mmHg, and normal saline was infused at a mean arterial pressure of 36 mmHg.

After 60 min of HS, 28 animals were randomly assigned into four groups by resuscitation method. The groups were as follows: HS without resuscitation (HS), HS resuscitation with shed-blood group (HSR), HS resuscitation with shed-blood and SFI (1 mL·kg^–1^) group (HSR-SFI), with eight animals in each of the three groups; and the sham operation group (Sham), which had four animals. Shen-fu injection(1 mL·kg^–1^) was administered in a single dose chosen based on the findings of previous experiments^15^.

Further, the pigs were administered a basal normal saline infusion of 10 mL·kg^–1^·h^–1^. They were euthanized with intravenous propofol (3 mg·kg^–1^), followed by potassium chloride (10 mL of 10 mol·L^–1^) 6 h after HS.

### Outcome measurements

#### Hemodynamic parameters

Hemodynamic parameters were continuously monitored and regularly recorded. Heart rate (HR), MAP and intrathoracic blood volume index (ITBVI) were measured at baseline, and at 1, 4 and 6 h post shock. Urine collected via a Foley catheter, and the urine output volumes were recorded per hour.

#### Blood samples

Venous blood was collected at baseline and at 1, 4 and 6 h following HS. The blood samples were used to measure serum creatinine (sCr), neutrophil gelatinase-associated lipocalin (NGAL), and cystatin C (CysC) levels, and all biomarkers were measured in duplicate by a single enzyme-linked immunosorbent assay (ELISA). Cytokine levels in plasma were analyzed at baseline and at 1, 4, and 6 h after HS. Tumor necrosis factor α (TNF-α), interleukin-1 beta (IL-1β), and interleukin-6 (IL-6) levels were determined by a quantitative sandwich ELISA using commercially available kits (RayBiotech, USA) specific for porcine cytokines.

#### Western blot

Part of the renal cortex was fixed in 4% paraformaldehyde, embedded in paraffin, and sliced to produce three sections per sample. BCL2-Associated X (BAX), B-cell lymphoma-2 (Bcl-2) and caspase-3 expression were measured using the streptavidin peroxidase method with immunohistochemistry kits (Santa Cruz Co., USA).

Five high-power fields were chosen and examined by light microscopy. The BAX, Bcl-2 and caspase-3 protein expressions in each section was represented by the integral optical density (IOD) and was analyzed using Image Pro Plus 6.0 software (Media Cybernetics, Inc., USA).

#### Immunohistochemical staining

Immunohistochemical staining was performed on the fixed kidney tissue slides using a standard protocol with primary antibodies, including monoclonal anti-BAX (50599-2-Ig,Proteintech), anti-Bcl-2 antibodies (12789-1-AP, Proteintech), and anti-caspase-3 (19677-1-AP, Proteintech) at 1:10,000 dilution (Cell Signaling Technology; Denver, USA) and secondary horseradish peroxidase-conjugated goat anti-mouse antibody at 1:1,000 dilution. The staining results were observed under optical microscopy (CX41; Olympus, Tokyo, Japan).

The IOD values of tissue sections in each group were measured by Image-Pro Plus 6.0 software (Media Cybernetics, Inc., Bethesda, MD, USA) after tissue images were captured under an optical microscope (400×). Five views were randomly selected to determine the positive IOD values, and the mean IOD values were defined as the relative expressions of BAX, Bcl-2, and caspase-3.

#### Renal tissue sampling

The kidney was surgically removed and preserved in 10% formaldehyde or 4% paraformaldehyde to observe pathologic changes under a light microscope. Kidney damage was scored by grading any glomerular, tubular, and interstitial change, based on a previous study^16^. The sum of the partial scores resulted in a final grade from zero to nine. Another portion of the kidney tissue was preserved to observe changes in ultra-microstructure under a transmission electron microscope. The pathologic evaluations were performed by an independent pathologist who has more than ten years of experience and was blinded to this study.

### Statistical analysis

Statistical analyses were performed with SPSS 19.0 software (SPSS, Chicago, IL, USA). Values are shown as mean ± standard deviation. Continuous variables were compared between groups. Repeated measures analysis of variance (ANOVA) was used to determine differences over time within groups, as appropriate. Least significant difference (LSD) method was used for paired groups comparisons. A two-sided p-value < 0.05 was considered statistically significant.

## Results

### Hemodynamic parameters

In the three experimental groups, the HRs were higher than that of the sham group one hour after reaching the target blood pressure of 40 mmHg (p < 0.001). There was no significant difference in HR between the HSR and HSR-SFI groups during resuscitation, either at 4 or 6 h. Mean aortic pressure in the HSR-SFI group was higher than that in the HSR group at 4 and 6 h post shock (p_4h_ = 0.040,p_6h_ = 0.048). No significant differences in ITBVI between the HSR and HRS-SFI groups during the experimental protocol were found (Table 1).

**Table 1:** Comparison of hemodynamic indexes and renal function in each group.

Group		Period			
		Baseline	1 hour	4 hours	6 hours
**HR (bpm)**					
	Sham (n = 4)	116.25 ± 16.17	117.25 ± 3.30	115.50 ± 9.33	106.75 ± 10.97
	HS (n = 8)	127.63 ± 25.35	200.25 ± 16.54^b^	224.75 ± 9.82^b^	222.75 ± 12.96^b^
	HSR (n = 8)	126.00 ± 25.84	213.25 ± 29.54	181.13 ± 19.87	184.75 ± 20.15
	HSR-SFI (n = 8)	118.38 ± 30.91	216.63 ± 27.81	191.63 ± 14.89	189.38 ± 34.13
**MAP (mmHg)**					
	Sham	111.50 ± 13.77	111.50 ± 7.14	109.25 ± 7.09	103.50 ± 11.73
	HS	110.88 ± 9.82	40.13 ± 2.03^b^	69.13 ± 9.73^b^	55.13 ± 9.96^b^
	HSR	112.63 ± 14.70	39.75 ± 1.67	94.88 ± 8.66	105.13 ± 7.20
	HSR-SFI	102.63 ± 19.66	40.13 ± 2.03	104.75 ± 9.54^c^	114.50 ± 9.74^c^
**ITBVI (mL·m^–2^)**					
	Sham	604.00 ± 120.72	572.25 ± 33.09	593.00 ± 73.23	497.00 ± 72.26
	HS	606.75 ± 136.92	322.75 ± 93.88^b^	345.63 ± 110.15^a^	347.00 ± 108.54^a^
	HSR	599.75 ± 150.08	383.75 ± 73.61	708.25 ± 152.94	621.25 ± 67.86
	HSR-SFI	598.88 ± 115.78	430.88 ± 120.01	634.13 ± 128.05	573.88 ± 51.94
**UV/per (mL**·**hour^–1^)**					
	Sham	94.38 ± 10.43	95.88 ± 8.11	103.00 ± 8.08	97.75 ± 8.73
	HS	86.41 ± 13.15	9.50 ± 4.34^b^	8.88 ± 6.42^b^	6.88 ± 7.74^b^
	HSR	91.16 ± 8.19	13.88 ± 7.00	64.38 ± 11.44	77.50 ± 9.49
	HSR-SFI	92.66 ± 6.82	13.00 ± 4.72	61.13 ± 11.41	92.63 ± 11.65^c^
**sCr** (**umol**·**L^–1^)**					
	Sham	101.03 ± 4.44	96.15 ± 8.72	98.73 ± 10.28	92.20 ± 8.55
	HS	96.13 ± 7.73	119.30 ± 12.49^a^	178.04 ± 30.55^a^	358.20 ± 59.64^b^
	HSR	98.40 ± 6.78	121.81 ± 14.41^a^	173.96 ± 38.11	220.69 ± 54.97
	HSR-SFI	99.69 ± 4.75	127.83 ± 23.80^a^	169.20 ± 45.23	169.20 ± 45.23^c^
**NGAL (ng**·**mL^–1^)**					
	Sham	1.49 ± 0.40	1.34 ± 0.27	1.45 ± 0.36	1.38 ± 0.36
	HS	1.46 ± 0.43	1.50 ± 0.33	5.08 ± 0.96^d^	9.12 ± 0.90^d^
	HSR	1.54 ± 0.56	1.77 ± 0.40	4.85 ± 0.89	7.43 ± 1.46
	HSR-SFI	1.57 ± 0.51	1.93 ± 0.51	4.49 ± 0.69	5.40 ± 1.35^c^
**CysC (ug**·L**^–1^)**					
	Sham	535.75 ± 44.78	507.30 ± 59.80	498.75 ± 22.27	527.28 ± 41.77
	HS	544.10 ± 39.91	531.59 ± 34.21	665.34 ± 66.79^b^	749.58 ± 67.47^b^
	HSR	532.59 ± 46.44	564.75 ± 45.64	593.62 ± 47.78	664.90 ± 68.23
	HSR-SFI	524.20 ± 29.39	539.11 ± 27.28	573.44 ± 28.95	577.30 ± 37.17^c^

Values are mean ± SE. ap < 0.05 vs. sham; bp < 0.001 vs. sham; cp < 0.05 vs. HSR; d p < 0.001 vs. HSR. Sham: the sham operation group, HS: the hemorrhagic shock group, HSR: the hemorrhagic shock and resuscitation group, HSR-SFI: the hemorrhagic shock and Shen-fu injection resuscitation group. HR: heart rate, MAP: mean arterial pressure, ITBVI: intrathoracic blood volume index, UV: urine volume, sCr: serum creatinine, NGAL: neutrophil gelatinase associated lipocalin, CysC: cystatin C.

### Renal function and biomarkers of AKI

One hour post shock, the urine volume per hour in the three experimental groups was significantly less than that in the sham group (p < 0.001). Six hours post-shock, the urine output per hour in the HSR-SFI group was higher than in the HSR group (p = 0.004). Additionally, at 6 h post shock, the sCr level in the HSR-SFI group was lower than that in the HSR group (p = 0.037) (Table 1).

Levels of NGAL and CysC in the HS group were higher than those in the sham group at 1 h post shock. In the HSR-SFI groups, the biomarkers were lower in the two resuscitation groups at 6 h post shock after (NGAL: p_6h_ = 0.002; CysC: p_6h_ = 0.006).

### Plasma cytokine levels

**Figure 1:**
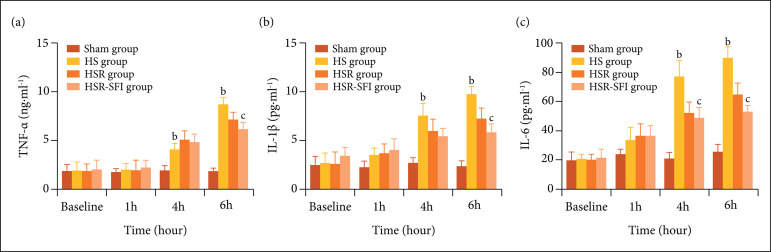
The plasma cytokine levels in four groups: a,p < 0.05 vs. sham; b,p < 0.001 vs. sham; c,p < 0.05 vs. HSR; d,p < 0.001 vs. HSR. **(a)** Tumor necrosis factor α; **(b)** Interleukin-1 beta; **(c)** Interleukin-6. At 4 and 6 h after shock, all cytokine levels (TNF-α, IL-1β and IL-6) in HS group were significantly higher than those in Sham group (p < 0.001). And at 6 h after shock, the cytokine levels in HSR-SFI groups were lower than those in HSR groups (pTNF-α = 0.0170.009; pIL-1β = 0.0160.005; pIL-6 = 0.0030.001). Sham: the sham operation group, HS: the hemorrhagic shock group, HSR: the hemorrhagic shock and resuscitation group, HSR-SFI: the hemorrhagic shock and Shen-fu injection resuscitation group.

At the 4 and 6 h intervals post shock, cytokine levels for TNF-α, IL-1β, and IL-6 in the HS group were significantly higher than those in the sham group (p < 0.001). Six hours post shock, the cytokine levels in the HSR-SFI groups were lower than those in HSR groups (p_TNF-α_ = 0.009; p_IL-1β_ = 0.005; p_IL-6_ = 0.001) (Fig. 1).

### Protein expression in the apoptotic pathway

**Figure 2:**
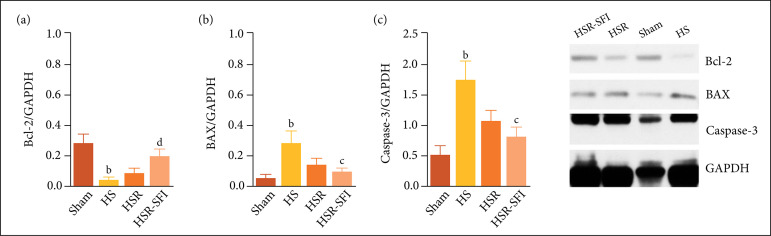
The expression of **(a)** Bcl-2, **(b)** BAX and **(c)** caspase-3 proteins. a,p < 0.05 vs. sham; b,p < 0.001 vs. sham; c,p < 0.05 vs. HSR; d,p < 0.001 vs. HSR compared with the Sham groups, Bcl-2 expression was significantly decreased, and BAX expression was increased in the HS groups (p < 0.001). Correspondingly, caspase-3 expression in the HS groups was higher than that in the sham groups (p < 0.001). Between the two resuscitation groups, Bcl-2 expression was significantly increased in the HSR-SFI groups (p < 0.001), and BAX expression was slightly reduced (p = 0.038). Caspase-3 expression in the HSR-SFI groups was lower than that in the HSR groups (p = 0.012). Sham: the sham operation group, HS: the hemorrhagic shock group, HSR: the hemorrhagic shock and resuscitation group, HSR-SFI: the hemorrhagic shock and Shen-fu injection resuscitation group.

After Western blot analysis, compared to the sham group, the expressions of Bcl-2 and BAX were significantly increased in the HS groups (p < 0.001). Correspondingly, caspase-3 expression in the HS groups was higher than that in the sham groups (p < 0.001). Between the two resuscitation groups, Bcl-2 expression was significantly increased (p < 0.001) and BAX expression was slightly reduced (p = 0.038) in the HSR-SFI groups. caspase-3 expression in the HSR-SFI groups was lower than that in the HSR groups (p = 0.012) (Fig. 2).

### Immunohistochemical staining

**Figure 3:**
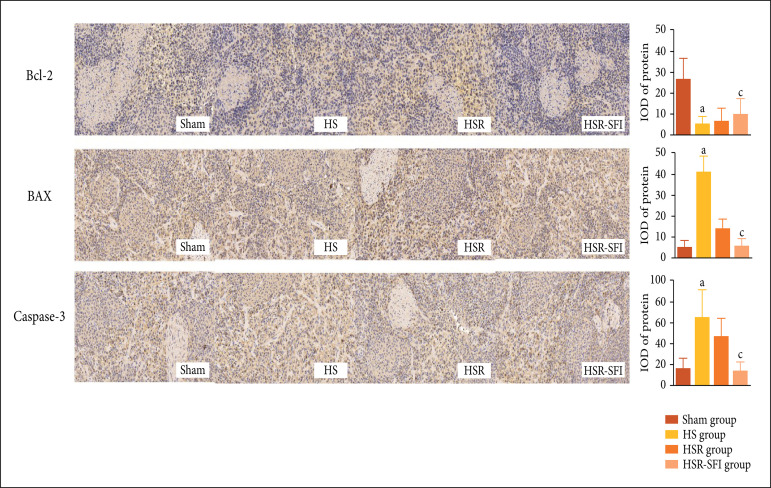
The expression of Bcl-2, BAX and caspase-3 proteins in four groups. a,p < 0.05 vs. sham; b,p < 0.001 vs. sham; c,p < 0.05 vs. HSR; d,p < 0.001 vs. HSR. Compared with sham group, the positive expression of Bcl-2 protein was lower(p = 0.032) and BAX protein was higher (p = 0.003) in HS group. Accordingly, the positive expression of caspase-3 protein in HS group was higher than that in sham group (p = 0.034). In the two resuscitation groups, the positive expression of Bcl-2 in HSR-SFI group was higher (p = 0.031), and the positive expression of BAX and caspase-3 proteins were lower (p_BAX_ = 0.041, p_caspase-3_ = 0.026). Sham: the sham operation group, HS: the hemorrhagic shock group, HSR: the hemorrhagic shock and resuscitation group, HSR-SFI: the hemorrhagic shock and Shen-fu injection resuscitation group.

The positive expression of an apoptosis-related protein in each group was observed. The expression of Bcl-2 protein was lower (p = 0.032) and that of BAX was higher (p = 0.003) in the HS group than in the sham group. The expression of caspase-3 in the HS group was higher than that in the sham group (p = 0.034). In the two resuscitation groups, the expression of Bcl-2 in the HSR-SFI group was higher (p = 0.031) and that of BAX and caspase-3 were lower (p_BAX_ = 0.041,p_caspase-3_ = 0.026) (Fig. 3).

### Renal histopathology

**Figure 4:**
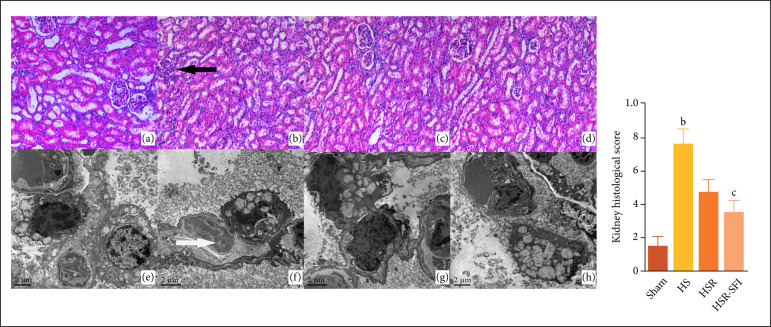
The comparison of renal pathological results in four groups. HE staining and light microscopy findings: (a) Sham group; (b) HS group; (c) HSR group; (d) HSR-SFI group. Original magnification: 200×. Transmission electron microscopy results: **(e)** Sham group; **(f)** HS group; **(g)** HSR group; **(h)** HSR-SFI group. Original magnification: 2000×. In the HS groups, the results of light microscopy showed that the glomerular capillaries are closed, the whole glomerulus becomes smaller, and the endothelial cells of the glomerulus and the outer poetasters are in a state of pyknosis (black arrow). The renal microcystitis was widened, and there were some fine particles in the cavity. The epithelium of the proximal convoluted tubules is slightly lower than that of the normal ones; the intercellular boundary is not clear. A fine granular substance is scattered in the lumen. In the HS groups, the microvilli on the free surface of the proximal convoluted tubules were incomplete, the mitochondria in the epithelial cells were significantly expanded, the arrangement of mitochondria cristae was disordered, and even disappeared (white arrow). Owing to the expansion and deformation of mitochondria, the basal folds around them were disordered, and the lysosomes in the cells were increased. Autophagy was observed, and the deformed mitochondria were engulfed. a,p < 0.05 vs. sham; b,p < 0.001 vs. sham; c,p < 0.05 vs. HSR; d,p < 0.001 vs. HSR. Sham: the sham operation group, HS: the hemorrhagic shock group, HSR: the hemorrhagic shock and resuscitation group, HSR-SFI: the hemorrhagic shock and Shen-fu injection resuscitation group.

In the HS groups, the results of light microscopy showed that the glomerular capillaries are closed, the whole glomerulus becomes smaller, and the endothelial cells of the glomerulus and the outer poetasters are in a state of pyknosis (Fig. 4, indicated by a black arrow). The renal microcystitis was widened, and there were some fine particles in the cavity. The epithelium of the proximal convoluted tubules is slightly lower than that of the normal ones; the intercellular boundary is not clear. A fine granular substance is scattered in the lumen.

Electron microscopy revealed that in the HS groups the microvilli on the free surface of the proximal convoluted tubules were incomplete, the mitochondria in the epithelial cells were significantly expanded, the arrangement of mitochondria cristae was disordered, and even disappeared (indicated by a white arrow). Owing to the expansion and deformation of mitochondria, the basal folds around them were disordered, and the lysosomes in the cells were increased. Autophagy was observed, and the deformed mitochondria were engulfed.

The histopathologic score in the HS group was significantly higher than that in the sham group(p < 0.001). Of the two resuscitation groups, the HSR-SFI group had a lower histopathologic score (p = 0.004).

## Discussion

This study confirmed that SFI reduces the risk of AKI due to hemorrhagic shock. Compared with the HSR group, the HSR-SF group produced more urine, had lower sCr levels, and lower plasma NGAL and CysC levels 4 and 6 h post shock, and their histopathologic scores were lower. Different from previous studies, according to these results, the protective effect of SFI on renal function not only comes from improving hemodynamics, but also may be related to attenuating systemic inflammatory response, and reducing apoptosis of renal tubular epithelial cells by regulating Bcl-2, BAX, and caspase-3 expressions.

According to previous studies, the AKI pathology during hemorrhagic shock and resuscitation is complex. In the early stage, AKI is mostly a function of trauma, hemorrhagic shock, and rhabdomyolysis. Later, as the injury progresses, it may relate more to a systemic inflammatory response, oxidative stress, and abdominal hypertension^17^.

In hemorrhagic shock, when the arterial pressure reaches the lower limit of autoregulation, renal blood flow decreases. Meanwhile, the increase of sympathetic activity and the release of renin and angiotensin lead to renal vasoconstriction, which worsens renal hypoxia^18^. No recognized optimal MAP exists for preserving renal function during resuscitation that follows hemorrhagic shock. However, when hemorrhaging is stopped, arterial pressure must be improved to optimize renal perfusion in case of vasoplegic shock^17^.

The results suggest that, compared with the standard-transfusion resuscitation, low-dose SFI and blood transfusion may increase MAP. Moreover, by comparing ITBVI between the two resuscitation groups, low-dose SFI did not increase the risk of pulmonary edema, which is a common complication in fluid resuscitation of hemorrhagic shock^19^. This effect may relate to increased left ventricular ejection fraction and an improved hemodynamic index of the heart^20^^,^ 21. A higher MAP adequately perfuses the kidney and protects its function, which subsequently increases urine output.

Hemorrhage often precipitates systematic inflammatory response^22^, because it stimulates the immune system, which increases inflammation via secondary-messengers, changes in gene expression, and neutrophil activation^23^. The sustained and exacerbated inflammatory response may be deleterious to renal function following hemorrhagic injury. In these results, the cytokine levels of the HS group at 4 and 6 h post shock were significantly higher than those of the sham operation group. In the two resuscitation groups, cytokine levels were lower in the HSR-SF group. This difference suggests that SFI can reduce serum inflammatory mediator levels, including TNF-α, IL-6, and IL-1β after HS and can attenuate excessive inflammatory responses.

The inflammasome-mediated, pro-inflammatory mediator releases contribute to the initiation, enhancement, and propagation of inflammation after trauma^24^, which promotes a further vascular endothelial barrier and parenchymal tissue damage^25^. This suggests that lower cytokine levels may reduce renal tissue damage post hemorrhage.

The relationship between apoptosis of the renal tubular epithelial cells and AKI is evident in studies on renal ischemia^26^^,^ 27. The mitochondrial pathway, as the “intrinsic” pathway of apoptosis^28^, controlled by the Bcl-2 protein family, activates caspase-3 when damaged mitochondria release cytochrome c in response to stress. BAX is a pro-apoptotic factor that participates in the mitochondrial apoptotic pathway. The Bcl-2, an antiapoptotic factor, can inhibit the function of BAX^29^. The balance between Bcl-2 and BAX determines whether the intrinsic apoptosis pathway is initiated^30^.

The results show that SFI likely reduces the apoptosis of renal tubular epithelial cells by reducing the expression of BAX, increasing the expression of Bcl-2, and inhibiting the expression of caspase-3. Similar findings have been reported in studies examining cardiac and lung tissues in a porcine model for cardiac arrest^6^^,^ 31 and in studies using a rat model to understand myocardial ischemia-reperfusion injury^32^.

## Limitations

This study was focused on the functional protection of a single organ and did not reflect long-term outcomes. Consequently, it is not possible to infer that SFI can reduce the mortality of porcine HS model. This study did not compare outcomes to those of vasoactive drug interventions, such as norepinephrine or vasopressin, so it is not possible to determine if SFI exerts better protective effects on the kidney than other interventions. The animal experiments differ in setting, the injury was intentionally inflicted in a controlled environment, and the study setting differs from the clinical environment. Further research is needed to recommend a clinical protocol.

## Conclusion

Shen-fu injection may reduce the risk of AKI following hemorrhagic shock by attenuating systemic inflammatory responses, and regulating the expression of apoptosis-related proteins.
